# The role of chromatin modifications in somatic embryogenesis in plants

**DOI:** 10.3389/fpls.2015.00635

**Published:** 2015-08-18

**Authors:** Clelia De-la-Peña, Geovanny I. Nic-Can, Rosa M. Galaz-Ávalos, Randy Avilez-Montalvo, Víctor M. Loyola-Vargas

**Affiliations:** ^1^Unidad de Biotecnología, Centro de Investigación Científica de Yucatán, MéridaMexico; ^2^Facultad de Ingeniería Química, Campus de Ciencias Exactas e Ingeniería, Universidad Autónoma de Yucatán, MéridaMexico; ^3^Unidad de Bioquímica y Biología Molecular de Plantas, Centro de Investigación Científica de Yucatán, MéridaMexico

**Keywords:** DNA methylation, epigenetics, histone modification, somaclonal variation, somatic embryogenesis

## Abstract

Somatic embryogenesis (SE) is a powerful tool for plant genetic improvement when used in combination with traditional agricultural techniques, and it is also an important technique to understand the different processes that occur during the development of plant embryogenesis. SE onset depends on a complex network of interactions among plant growth regulators, mainly auxins and cytokinins, during the proembryogenic early stages, and ethylene and gibberellic and abscisic acids later in the development of the somatic embryos. These growth regulators control spatial and temporal regulation of multiple genes in order to initiate change in the genetic program of somatic cells, as well as moderating the transition between embryo developmental stages. In recent years, epigenetic mechanisms have emerged as critical factors during SE. Some early reports indicate that auxins and *in vitro* conditions modify the levels of DNA methylation in embryogenic cells. The changes in DNA methylation patterns are associated with the regulation of several genes involved in SE, such as *WUS*, *BBM1, LEC*, and several others. In this review, we highlight the more recent discoveries in the understanding of the role of epigenetic regulation of SE. In addition, we include a survey of different approaches to the study of SE, and new opportunities to focus SE studies.

## Introduction

Somatic embryogenesis (SE) is a powerful tool for plant genetic improvement when it is used in combination with traditional agricultural techniques (Loyola-Vargas et al., 2008). SE onset depends on a complex network of interactions among plant growth regulators, mainly auxins and cytokinins, during the proembryogenic early stages, and ethylene and gibberellic and abscisic acids later in the development of somatic embryos. These growth regulators control the spatial and temporal expression of multiple genes in order to initiate change in the genetic program of the somatic cells, as well as the transition between embryo developmental stages (Fehér, 2015).

Plants as well as animals have sophisticated mechanisms to regulate cellular division, development and growth (Albert and Peters, 2009; Gonzalo, 2010; Wollmann and Berger, 2012; Nic-Can et al., 2013). Chromatin organization allows the expression or repression of genes depending on the degree of its compaction in a specific locus (Schones and Zhao, 2008; Tamaru, 2010). This chromatin compaction results from two main processes, histone modification and DNA methylation. Both are present in plants and animals; however, DNA methylation in plants is more complex than in animals. Other processes controlled by DNA methylation are the transcription of invading and mobile DNA elements, such as viruses, transgenes, transposons, and retroelements (Law and Jacobsen, 2010; Feng and Jacobsen, 2011).

DNA methylation is carried out by the addition of a methyl group at the 5′position of the pyrimidine ring of cytosine in the DNA (5mC). In animals, this methylation occurs in a cytosine that is adjacent to a guanine (CpG) (Vanyushin, 1984). However, methylation in plants is not always in the CpG islands (Gruenbaum et al., 1981; Belanger and Hepburn, 1990); it can also be done in CpHpG and CpHpHp (where H is any nucleotide except G; Finnegan et al., 1998; Feng et al., 2010).

During early embryo development, DNA methylation is continually changing in order to satisfy the cell requirements. In animals, *de novo* methylation is necessary for embryo implantation (Monk et al., 1987); if this methylation is not achieved, the survival of the embryo could be compromised (Okano et al., 1999). In plants, which form an embryo without egg fertilization, the dynamic of the methylation depends on embryo development (Nic-Can et al., 2013), as well as the species (Nic-Can and De-la-Peña, 2014). Plants are able to survive larger reductions in genomic 5mC than animals. This phenomenon is very relevant, since DNA demethylation produces an important increase in the rates of transposon insertion (Hirochika et al., 2000; Singer et al., 2001; Kato et al., 2003; Tsukahara et al., 2009). On the other hand, the exposure to *in vitro* culture conditions produces epigenetic variation at several levels (Kaeppler and Phillips, 1993; Smykal et al., 2007; Valledor et al., 2007; Miguel and Marum, 2011; De-la-Peña et al., 2012; for a review see Neelakandan and Wang, 2012; Us-Camas et al., 2014).

## Methyltransferases

**FIGURE 1:**
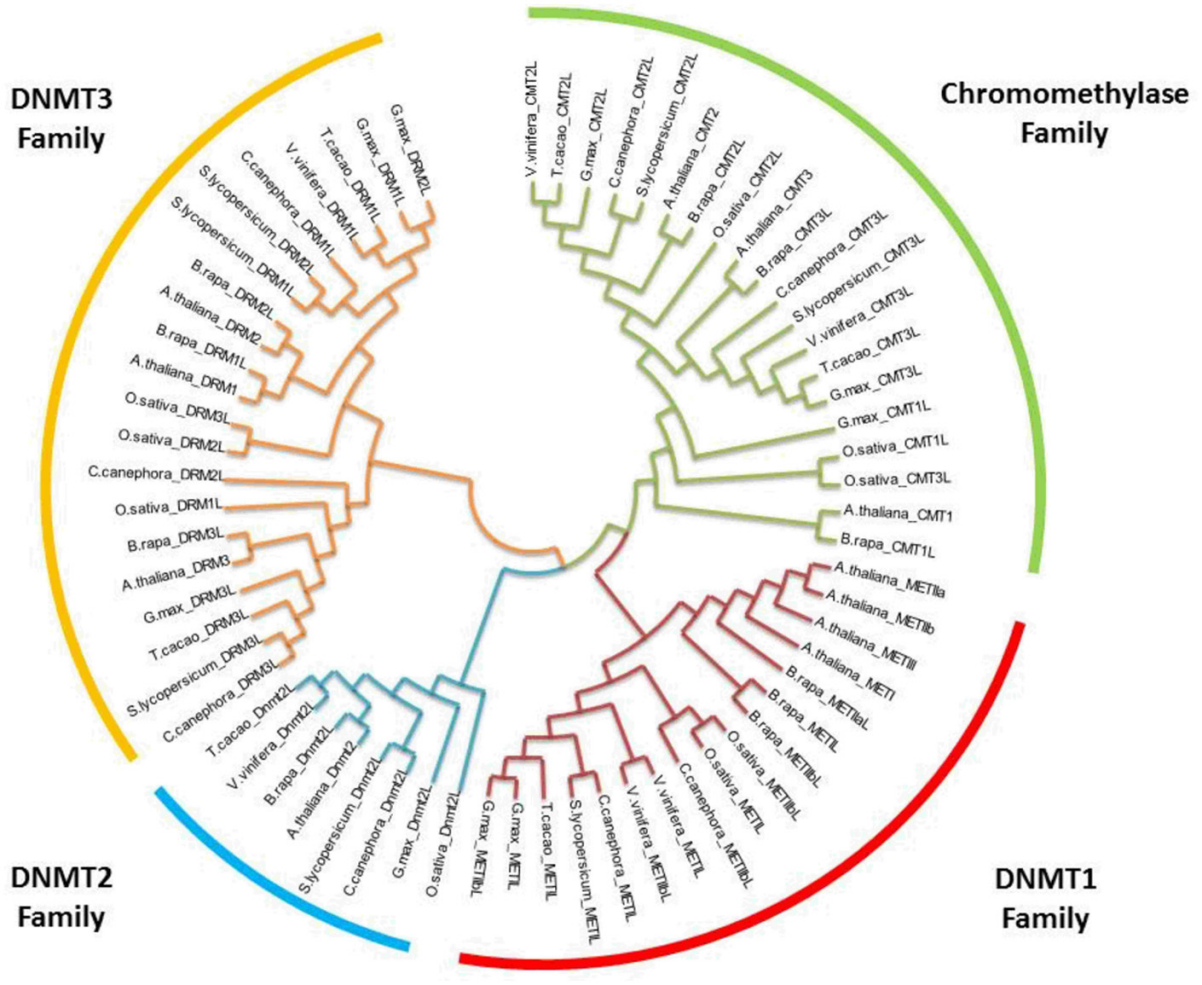
**Phylogenetic relationships of orthologous DNA (cytosine-5-)-MTases among various plants**. The evolutionary history was inferred by using the maximum likelihood method based on the Tamura-Nei model (Tamura and Nei, 1993). Bootstrap analyses consisted of 500 replicates. The analysis involved 66 nucleotide sequences. All positions with less than 95% site coverage were eliminated. Evolutionary analyses were conducted in MEGA6 (Tamura et al., 2013). *Oryza sativa* was used as the outgroup.

Methylation of DNA is catalyzed by a set of enzymes named DNA (cytosine-5-)-methyltransferase (DCMTases; EC 2.1.1.37). With the exclusion of fungal enzymes, and based on the sequence homology within their C-terminal catalytic domains, most DCMTases can be grouped into four distinct families (Grace Goll and Bestor, 2005). Plants have all four classes of DCMTases, while other eukaryotic organisms have only two or three classes. In plants, these groups of DCMTases are named DNA methyltransferase1 (MET1), domains rearranged methyltransferase (DRM), DNA nucleotide methyltransferase2 (DNMT2) and chromomethylase3 (CMT3).This last group appears to be unique to plants. There is significant variability in the types and numbers of DCMTases in plants (**Table 1**; **Figure 1**); e.g., *Arabidopsis thaliana* has 11, *Glycine max* has nine, *Coffea canephora* has eight, and *Vitis vinifera* and *Theo broma cacao* have six.

**Table 1:** Genes codifying for methyltransferases in some genome plants.

Species	Family	Methyltransferases			
		MET1	DNMT2	DRM	CMT3
		
		Substrate specificity			
		Maintenance CG&CHG	Broader specificity	*De novo* CG&CHG Maintenance CHH	Maintenance CHG&CHH
*A. thaliana*	*Brassicaceae*	4	1	3	3
*B. rapa*	*Brassicaceae*	3	1	3	3
*S. lycopersicum*	*Solanaceae*	1	1	3	2
*V. vinifera*	*Vitaceae*	2	1	1	2
*C. canephora*	*Rubiaceae*	2	1	3	2
*G. max*	*Leguminosae*	2	1	3	3
*T. cacao*	*Malvaceae*	1	1	2	2
*O. sativa*	*Poaceae*	2	1	3	3

*The analysis for the presence of MTases was carried out using the complete sequence for *Coffea canephora* from Coffee Genome Hub (http://coffee-genome.org/), for *Arabidopsis thaliana* from TAIR10 (http://www.arabidopsis.org/) and for *Solanum lycopersicum*, *Vitis vinífera*, *Theobroma cacao*, *Brassica rapa*, *Glycine max*, and *Oryza sativa* from PHYTOZOME v10.2 (http://phytozome.jgi.doe.gov/pz/portal.html). We took into account all of the sequences that proved to contain more than one of the following functional annotations: PFAM/PF00145C-5/cytosine-specific DNA methylase, DNA (cytosine-5-)-methyltransferase [EC:2.1.1.37] and (GO:0008168) [QuickGo from European Bioinformatics Institute].*

Using sequence similarity to Dnmt1 [the plant homolog of mammalian DNA (cytosine-5) methyltransferase 1], a DCMTase named MET1 was identified and cloned in *A. thaliana* (Jean Finnegan and Dennis, 1993). MET1 catalyzes the maintenance methylation of the CG islands in the heterochromatin (Cao and Jacobsen, 2002; Cokus et al., 2008; Lister et al., 2008), but may also play a role in *de novo* methylation (Finnegan and Kovac, 2000); DMR and CMT3 are in charge of the maintenance methylation of CHG and CHH isles (Lindroth et al., 2001; Law and Jacobsen, 2010; Du et al., 2012; Köhler et al., 2012), and DMR also methylates *de novo* CG, CHG, and CHH. It is dependent on RNAi-like machinery (Law and Jacobsen, 2010).

Methyltransferase enzymes have important motif characteristics to facilitate their main functions. The crystallization of the methyltransferase domain of *Nt*DMR from tobacco shows a classic class I methyltransferase fold. The enzyme forms a homodimer with the dimer interface mimicking the mammalian Dnmt3a-Dnmt3L heterodimer interface (Zhong et al., 2014). This is very interesting because this family of enzymes shows a strong conservation of the catalytic motifs in their C-terminal domains with mammalian Dnmt3A and Dnmt3B proteins (**Figure 1**) (Grace Goll and Bestor, 2005).

Chromomethylases are unique to flowering plants, and were identified by Henikoff and Comai (1998). These enzymes possess a chromodomain between motifs II and IV (Henikoff and Comai, 1998) and keep the eight conserved motifs characteristic of eukaryote cytosine methyltransferases (Finnegan and Kovac, 2000).

The sequence alignments and comparison of DCMTases present in the genome of eight plants, the genomes of which have been sequenced, was carried out (**Figure 1**). The cladogram reveals a clear division among the four groups of DCMTases present in plants. The family DNMT1 shares 46–60% similarity between *A. thaliana* and *C. canephora*. For the families DNMT2, DNMT3 and chromomethylase, these values are 65, 36–52%, and 53–60%, respectively, for both species.

**FIGURE 2:**
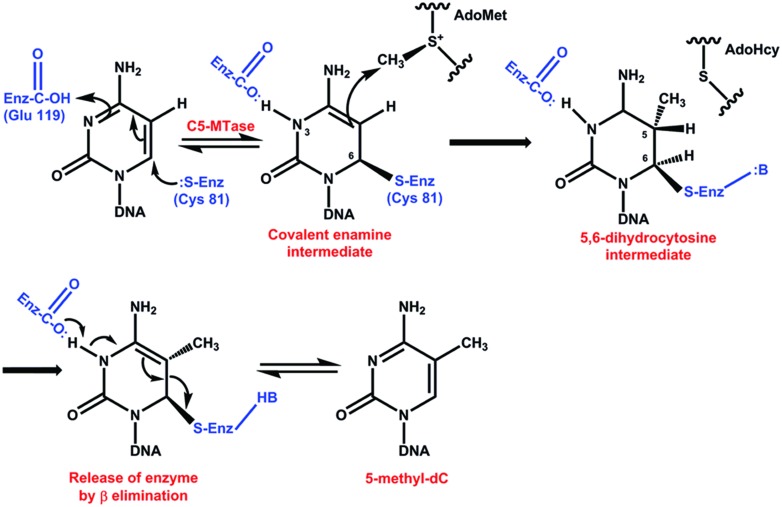
**Catalytic mechanism of DNA methyltransferases**. The reaction initiates with a nucleophilic attack on carbon 6 of cytosine in DNA. This nucleophilic attack activates an original inert carbon 5. Abstraction of the proton at the C5 position followed by β elimination allows reformation of the C5–C6 double bond and releases the active enzyme and DNA with a methylated cytosine (Santi et al., 1983, 1984; Wu and Santi, 1987; Klimasauskas et al., 1994; Peräkylä, 1998; Liutkeviciute et al., 2011).

A number of chemical and enzymatic studies show that the catalytic mechanism of DCMTases initiates with a nucleophilic attack of a conserved cysteine-81 (Cys) from the active site of the enzyme on carbon 6 (C6) of cytosine in DNA and generates a covalent enzyme DNA intermediate (**Figure 2**).This nucleophilic attack activates an original inert carbon 5 (C5), and the flow of electrons to C5 produces a negative charge and leads to an attack on the methyl group of S-adenosyl-L-methionine (electrophile). The nucleophilic attack increases the pK of the N3 of the cytosine and this nitrogen is protonated. This part of the reaction takes place with the Glu119 of the enzyme active site. Abstraction of the proton at the C5 position followed by β elimination allows reformation of the C5–C6 double bond and releases the active enzyme and DNA with a methylated cytosine (**Figure 2**) (Santi et al., 1983, 1984; Wu and Santi, 1987; Klimasauskas et al., 1994; Peräkylä, 1998; Liutkeviciute et al., 2011).

## Inhibitors

**FIGURE 3:**
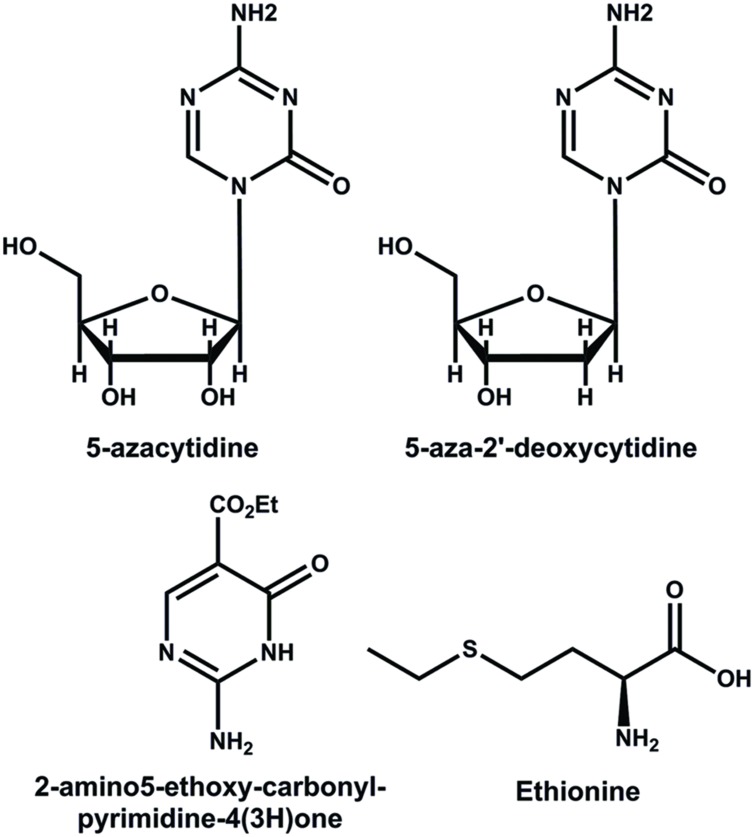
**Structures of some inhibitors of the methylation of DNA**.

Methyltransferases and their function in DNA methylation can be modified by a set of compounds that interferes in different steps of the methylation process (Goffin and Eisenhauer, 2002). The pyrimidine analogs 5-azacytidine (azaC) and the 5-aza-2′-deoxicytidine (decitabine) are cytosine analogs that, instead of the carbon atom at position 5, have a nitrogen atom (**Figure 3**) (Jones and Taylor, 1980). During the replication of DNA, (Lübbert, 2000), these compounds can be incorporated into the DNA, avoiding the methylation of DNA and resulting in a general genome hypomethylation (Santi et al., 1983). The 2-amino5-ethoxy-carbonyl-pyrimidine-4(3H)-one is a pyrimidine analog that also inhibits the methylation of DNA (**Figure 3**) (Raugei et al., 1981). Another compound employed in epigenetic studies is ethionine (2-amino-4-ethylsulfanylbutyric acid) (**Figure 3**), used by cells to produce *S*-adenosyl-L-methionine, which functions as a competitive inhibitor of DNA methylation (Cox and Irving, 1977).

**FIGURE 4:**
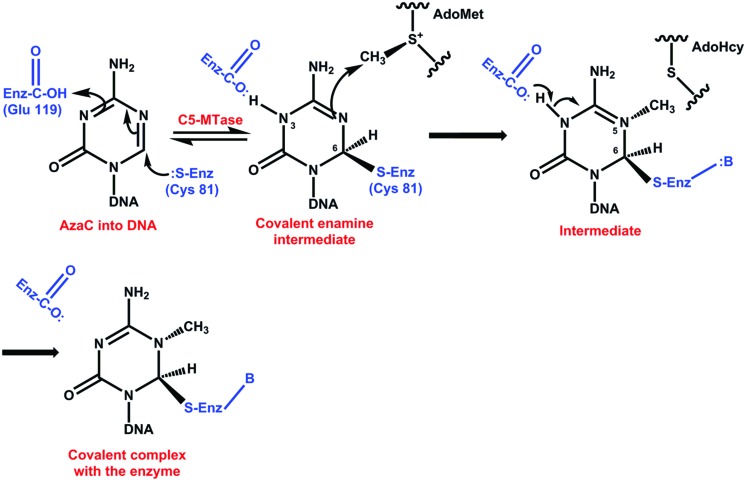
**Catalytic mechanism of the inhibition of DNA methylation by 5-azacytidine**. After the formation of C6 and the enzyme, slow methyl transfer takes place; however, the absence of hydrogen at C5 avoids the β elimination and the enzyme remains attached to the complex (Santi et al., 1983, 1984; Santini et al., 2001).

It has been suggested that the inhibitory mechanism of DNA methylation by pyrimidine analogs could be through the formation of a covalent bond between a catalytic nucleophile site of the DCMTases and the reactive 6 position of azaC that has replaced cytosine in DNA (Bouchard and Momparler, 1983; Santi et al., 1983; Jüttermann et al., 1994). The substitution of carbon by nitrogen at position 5 changes the reactivity of carbon at position 6, avoiding the reversibility of the bond between this carbon and a cysteine at the active site of the enzyme (**Figure 4**) (Santi et al., 1983, 1984). After repeated replication of cell cycles, the inhibitor depletes the cell of DCMTases, resulting in the hypomethylation of DNA (Santini et al., 2001). Another possible mechanism of action of these inhibitors would be through the damage to the structural stability of DNA (Lin et al., 1981; D’Incalci et al., 1985).

AzaC and decitabine can be also incorporated into DNA or RNA (Santini et al., 2001). AzaC is incorporated preferentially into RNA (Santini et al., 2001) and decitabine into DNA. The incorporation of azaC into RNA produces a ribosome malfunction and inhibits protein synthesis. All of these mechanisms have been studied in animal cells, but at present there has not been a study of plant cells in order to determine whether the inhibition mechanism is the same as that in animals.

## Techniques to Determine DNA Methylation

DNA methylation is an important and widely used regulatory process among higher organisms. This led to the development of precise and efficient methods to determine the genomic DNA methylation content, as well as the specific sites of methylation in order to elucidate its role in biological processes such as SE.

**FIGURE 5:**
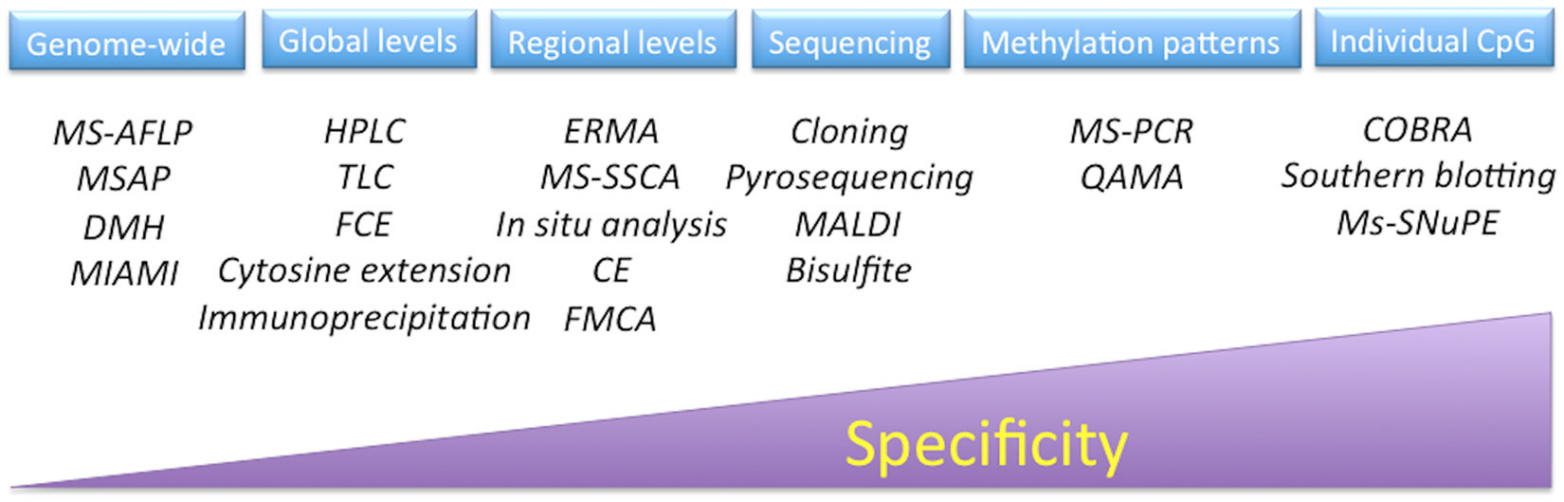
**Techniques to analyze DNA methylation**. MS-AFLP, methylation-sensitive amplified fragment length polymorphisms; MSAP, methylation-sensitive amplification polymorphism; DMH, differential methylation hybridization; MIAMI, microarray-based integrated analysis of methylation by isochizomers; HPLC, high-performance liquid chromatography; TLC, thin-layer chromatography; FCE, fluorescent capillary electrophoresis; ERMA, enzymatic regional methylation assay; MS-SSCA, methylation-sensitive-single strand conformation analysis; CE, capillary electrophoresis; FMCA, fluorescence melting curve analysis; MALDI, matrix-assisted laser desorption/ionization mass spectrometry; MS-PCR, methylation-sensitive polymerase chain reaction; QAMA, quantitative analysis of methylated alleles; COBRA, combined bisulfite restriction analysis; MS-SNuPE, methylation-sensitive-single nucleotide primer extension.

The methods for the determination of methylation levels in DNA can be divided into at least into six general groups: global DNA methylation, regional DNA methylation, genome-wide analysis, DNA methylation analysis by sequencing, detection of specific methylation patterns, and individual CpG analysis (**Figure 5**). Some of these have been applied to study the process of both SE and zygotic embryogenesis (El-Tantawy et al., 2014; Nic-Can and De-la-Peña, 2014; Wolny et al., 2014; Pérez et al., 2015b); however, it is necessary to expand current strategies in order to have a more precise understanding of these important processes. For a complete analysis of all of the techniques used to study chromatin modifications, see Tost (2009), Kovalchuk and Zemp (2010), Tollefsbo (2011), and Spillane and McKeown (2014).

## Analysis of DNA Methylation by Bisulfite Sequencing

**FIGURE 6:**
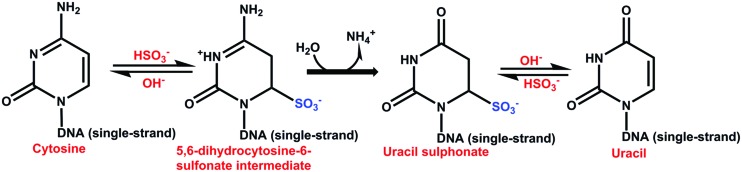
**Catalytic mechanism of the reaction of bisulfite with cytosine.** The sulphonation is favored at low pH and low temperature. The hydrolytic deamination of cytosin-6-sulphonate to uracil-6-sulphonate is irreversible. The desulphonation of uracil-6-sulphonate to uracil is reversible and is favored at high pH (Wang et al., 1980; Frommer et al., 1992; Hajkova et al., 2002).

The bisulfite genomic sequencing method (Frommer et al., 1992) is both qualitative and quantitative. This method is based on the conversion of cytosines in single-stranded DNA into uracil by sodium bisulfite, which is recognized as thymine in subsequent PCR amplification and sequencing. The 5mCs do not react to this transformation and remain cytosines, allowing 5mCs to be distinguished from unmethylated cytosines. The first step in this method is to denaturalize the double strand of DNA; this is followed by the sulphonation of the cytosine residues at the C-6 position, hydrolytic deamination at C-4 to produce uracil-sulphonate, and desulphonation under alkaline conditions (**Figure 6**) (Wang et al., 1980; Grigg, 1996; Rein et al., 1998; Hajkova et al., 2002). The 5mC is unreactive due to the inability of bisulfite to access the C-6 position. This method has been used to determine the methylation status at a specific locus of genes involved in the SE of *Daucus carota* (Shibukawa et al., 2009) and *C. arabica* (Bobadilla Landey et al., 2013). The bisulfite method has become the basis for other methods, such as methylation-sensitive single nucleotide primer extension (Ms-SNuPE), combined bisulfite restriction analysis (COBRA), methylation-specific PCR (MSP), and others that would be interesting to apply during the transition of somatic cells into embryogenic ones.

## Quantification of Global DNA Methylation

Global DNA methylation is frequently used to evaluate whether DNA methylation changes exist on a large scale during growth and development or if they are induced by different environmental signals in plants and animals. Global 5mC levels can be detected by several analytical techniques, such as reversed-phase high performance liquid chromatography (RP-HPLC), capillary electrophoresis, inductively mass coupled mass plasma spectrometry (ICP-MS), coupling liquid chromatography or gas chromatography, and electrospray ionization mass spectrometry (ESI-MS; Magaña Alcázar et al., 2008; Wrobel et al., 2009; Lopez Torres et al., 2011). Among these analytical techniques, RP-HPLC is the most common procedure. The level of 5mC is obtained through enzymatic digestion of DNA (DNAse, nuclease P1 and phosphatase alkaline) to obtain free deoxynucleosides, followed by chromatographic separation, where the suitable separation of deoxynucleosides must be ensured. The use of the HPLC technique to analyze DNA methylation has been effective at determining the methylation changes throughout the whole SE process in *C. canephora* (Nic-Can et al., 2013) and during the SE of *Castanea sativa* (Viejo et al., 2010).

## Methylation-Sensitive Amplified Polymorphism (MSAP)

Analysis of methylation-sensitive amplified polymorphism (MSAP) has been applied to several plants in order to identify genome-wide epigenetic variations. This technique is based on the use of a pair of methylation-sensitive restriction enzymes, HpaII and MspI, which are isoschizomers, as well as the use of EcoRI. Both enzymes recognize the same sequence, 5′-CCGG-3′; however, their action is affected by the methylation pattern at the inner or outer cytosine (Reyna-López et al., 1997). MSAP has proven to be an efficient method for detecting alterations in cytosine methylation in fixed genotypes (Li et al., 2008); this technique is relatively simple and has been often used to assess different systems of plant tissue cultures with the purpose of identifying genes under epigenetic control (Miguel and Marum, 2011). In addition, MSAP does not require a sequenced reference genome, but the scoring of MSAP data should be made carefully in order to determine the distribution of CpG methylation at the 5′-CCGG-3′sites through the genome (Abid et al., 2011). Xu et al. (2004) used this technique in *Rosa hybrida* cv. Carefree, and found that the demethylation of outer cytosines occurred at a high frequency during SE.

## *In Situ* Analysis of DNA Methylation

As the realization of the importance of DNA methylation to different biological processes is growing (Martienssen and Colot, 2001; Bruce et al., 2007; Amasino, 2010), the number and sensitivity of techniques to measure 5mC is also receiving more attention. Many techniques, such as MSAP (Baurens et al., 2008), HPLC (De-la-Peña et al., 2012), high-performance capillary electrophoresis (HPCE; Fraga et al., 2000; Meijón et al., 2009) and ELISA-based assays (Testillano et al., 2013) have been used effectively to measure global DNA methylation levels. However, some studies are applying a more sensitive spatial and temporal analysis to localize the precise distribution of 5mC (Meijón et al., 2009; Testillano et al., 2013).

*In situ* analysis using immunolocalization coupled to confocal microscopy in order to localize 5mC to an exact moment and cell have resolved many biological questions (Meijón et al., 2009; Kathiria and Kovalchuk, 2010; Testillano et al., 2013; Pérez et al., 2015b). This technique is designed to work with antibodies and a confocal microscope, and it is possible to detect the fluorescence signal with high sensitivity and good reproducibility (Kathiria and Kovalchuk, 2010). This technique has not been applied to the SE process, but it is clear that it would give interesting results about specific methylation sites during the transition from the globular to the heart stage.

## Epigenetics of Somatic Embryogenesis

In recent years, epigenetic mechanisms have emerged as critical factors during the induction of both somatic (Nic-Can and De-la-Peña, 2014) and zygotic embryogenesis (Nodine and Bartel, 2010). The modifications present during the induction of SE and development of somatic embryos include methylation of DNA, as well as histone modifications (**Table 2**). The methylation of DNA is essential in order for SE to succeed. This epigenetic mechanism during the induction of SE has been documented in at least 18 species from 12 families (**Table 2**). However, in only four of these species have the modifications in histones been determined (Nic-Can et al., 2013; Rodríguez-Sanz et al., 2014b; Pérez et al., 2015a; Wickramasuriya and Dunwell, 2015).

**Table 2:** DNA methylation and histone modifications during the induction of somatic embryogenesis and development of somatic embryos.

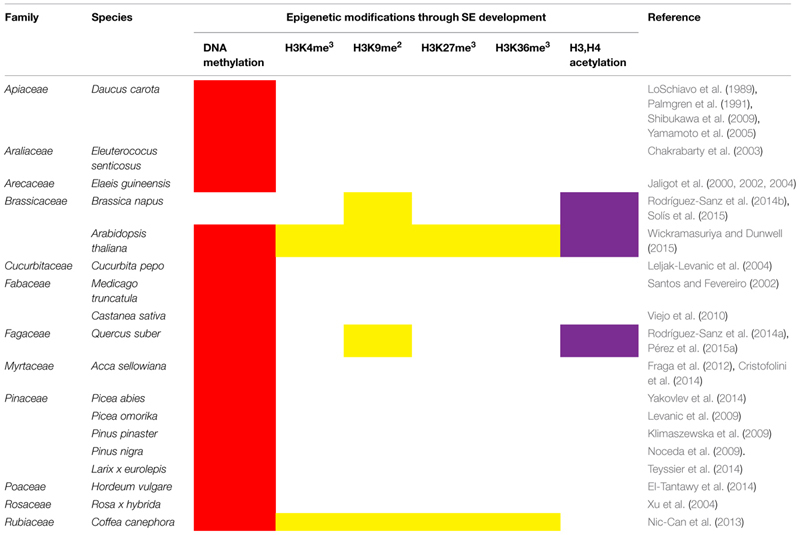

In general, the methylation of DNA is lower in the embryogenic tissues than in the non-embryogenic tissues. For instance, in Siberian ginseng (*Eleutercoccus senticosus*), the non-embryogenic calli showed higher DNA methylation in the sites 5′-CCGG-3′ (16.99%) than the embryogenic calli (11.20%) (Chakrabarty et al., 2003). A similar pattern has been determined in *Pinus nigra* Arn. ssp Austriaca, in which embryogenic lines showed low methylation levels (Noceda et al., 2009). Since the lowest level of DNA methylation is always found in the embryogenic cells (Palmgren et al., 1991), it is possible that DNA hypomethylation is involved in the signal that leads to the induction of SE. The transient expression of a carrot DNA methyltransferase gene, Met1-5, after the induction of SE by 2,4-dichlorophenoxiacetic acid (2,4-D) and before the formation of embryogenic cell clumps (Yamamoto et al., 2005), seems to support this theory. However, these studies are in contradiction with the finding from *P. pinaster*, where there are no differences in the amount of DNA methylation of embryogenic and non-embryogenic lines. The DNA methylation values are between 17.8 and 19.1%, with no significant difference (Klimaszewska et al., 2009). Nevertheless, the determination of total 5mC can lead to underestimating the methylation/demethylation of specific sites of DNA. To avoid this problem, it is necessary to use more reliable techniques, such as MSAP. Using a modification of this technique, Xu et al. (2004) were able to show that in *R. hybrida* cv. Carefree, the demethylation of outer cytosines occurred at a high frequency during SE. This indicates that besides the total 5mC quantification, it is necessary to evaluate specific methylation sites in order to have more complete information about DNA methylation not only for SE but also for other systems.

The study of the epigenetic changes during the induction of SE and the development of the somatic embryos is not an easy task. Several factors can affect the changes in the methylation pattern of DNA. Among these factors are the age of the cell lines, the genetic background of the explant, the presence/absence of growth regulators, the culture medium components, the physiological conditions of the explant, the temperature of incubation, molecules secreted by the explants and others. For instance, it was found in *D. carota* that the presence of low levels of 2,4-D (2.26 μM) favored a low level of 5mC (i.e., 16%), whereas a 10-fold increase in the concentration of 2,4-D increased the methylation levels to 45%. A similar concentration of different auxins, such as 1-naphthaleneacetic acid (NAA) and indole-3-acetic acid (IAA), only increased the level of DNA methylation to 23% (LoSchiavo et al., 1989). This suggests that DNA methylation is affected not only by the presence of auxins, but also by the type of the auxin used. DNA methylation is also affected by the nitrogen source (Leljak-Levanic et al., 2004). In *Cucurbita pepo* embryogenic lines, it was found that a low amount of NH_4_Cl present in the B_5_ medium is enough to produce the highest rate of DNA methylation in an auxin-free medium (Leljak-Levanic et al., 2004).

Since the discovery of SE, it has been found that the stage of development of some tissues is crucial for the production of somatic embryos (Loyola-Vargas et al., 2008). Recently, Viejo et al. (2010) found that the induction of SE from zygotic embryos of *C. sativa* depends on a decrease in DNA methylation of the original explant. It seems that the induction of SE occurs when a decrease in DNA methylation happens during the zygotic embryo maturation. It is possible that the genotypic dependence of the induction of SE is related to the epigenetic status of the explant.

In general, it appears that the hypomethylation of the explant is a prerequisite for a successful induction of SE. However, the mechanism by which this hypomethylation affects the induction of SE is unclear. Using the SE of carrot as a model, two laboratories have reported contradictory results. Yamamoto et al. (2005) showed that azaC down-regulated the expression of the transcription factor *CARROT-LEAFY COTYLEDON1* (*DcLEC1*) during morphogenesis of embryos from epidermal carrot cells, while Shibukawa et al. (2009) showed that it is the hypermethylation of a portion from -1,904 to -1,272 of the 5′-upstream region of the promoter of *DcLEC1* that actually down-regulates its expression. However, in both cases it is suggested that the expression of *DcLEC1* during carrot SE could be regulated by DNA methylation.

The use of pharmacological approaches in the study of metabolic pathways and their regulation has been very useful. The study of the epigenetic marks during the induction of SE and development of the somatic embryos is not an exception. The treatment of *Medicago truncatula* (Santos and Fevereiro, 2002) and *D. carota* (Yamamoto et al., 2005) embryogenic lines with azaC not only decreased the production of somatic embryos but also caused the loss of the SE. In the case of *M. truncatula*, it was found that the disruption of the SE response was due to an increase of demethylated rDNA(Santos and Fevereiro, 2002). However, in *D. carota* it was shown that the effects of azaC on SE depend on the embryogenic stage at which it is applied. Yamamoto et al. (2005) reported that this demethylating agent suppresses the SE if it is applied between 3 and 7 days after induction, but not if it is applied after day 7. These results suggest that certain levels of DNA methylation have to be maintained before the change from somatic cell to embryogenic cell. Similar to *D. carota*, the presence of azaC during the induction of SE in *C. canephora* decreased the DNA methylation and severely inhibited the embryogenic response when it was applied at both 7 and 14 days (Nic-Can et al., 2013). It was observed that the presence of azaC decreased the transcript levels of *LEC1* and *BABY BOOM1* (*BBM1*), impairing the embryogenic program. However, it was also observed that the SE response was improved and synchronized, even at a concentration of 20 μM of azaC, if it was added at 35 days after the embryogenic induction (Nic-Can et al., 2013). A similar reduction in DNA methylation has been observed in *P. omorika* after 1 week of exposure to azaC in the presence of 2,4-D and BA, with no effect on embryo development (Levanic et al., 2009). However, in *C. pepo* the use of azaC did not inhibit either the induction of SE or the amount of somatic embryos in the different developmental stages, compared with the controls without azaC (Leljak-Levanic et al., 2004). In *Acca sellowiana*, 200 μM of 2,4-D and 10 μM of azaC increased the formation of somatic embryos by more than 240% in accession 101 × 458, but not in accession 85. The same experiment, but using 50 μM of azaC, increased the formation of somatic embryos by more than 240% in accession 85 and decreased to 10% the formation of somatic embryos in accession 101 × 458 (Fraga et al., 2012). Meanwhile, the treatment of *P. pinaster* SE lines with 5 μM or greater of azaC reduced the growth of the cultures. However, DNA methylation remained around 18.7%. Furthermore, concentrations of 10 and 15 μM of azaC produced a slight increase in the number of mature somatic embryos (Klimaszewska et al., 2009). In *Brassica napus* and *Hordeum vulgare*, the incubation for 4 days with 2.5 μM of azaC increased embryo induction and modified the heterochromatin patterns (Solís et al., 2015). However, when the azaC treatment was longer, the number of embryos diminished.

It is known that stress can be an inducer of SE and this fact raises the question of whether/how both phenomena can share common signaling pathways (Yang and Zhang, 2010). The change in the genetic program from microspore to SE could be due to heat (*B. napus*, *Nicotiana tabacum*; Touraev et al., 1996; Zhao et al., 1996; Solís et al., 2015) or cold (*H. vulgare*; El-Tantawy et al., 2014). However, other stresses such as osmotic shock (Kamada et al., 1993; Cabrera-Ponce et al., 2015), water deficit (Patnaik et al., 2005; Meneses et al., 2010), temperature (Decout et al., 1994; Kamada et al., 1994), heavy metals (Ikeda-Iwai et al., 2003; Patnaik et al., 2005), wounding (Nolan et al., 2006), nutrient starvation (Fuentes-Cerda et al., 2001; Mihaljevic et al., 2011), culture medium dehydration (Jin et al., 2014), ultraviolet radiation, and pH (Pasternak et al., 2002; Potters et al., 2007) might also play a major role in somatic embryo induction.

The use of massive transcriptome sequence during the SE induction in *Zea mays* (Salvo et al., 2014), *Gossypium hirsutum* (Jin et al., 2014) and *A. thaliana* (Wickramasuriya and Dunwell, 2015) have revealed the close relationship between the signaling pathways leading to stress and morphogenic responses. Both processes share the expression of many stress-morphogenic-related genes. These findings have led to the question of whether SE is a stress response of plants in order to survive extreme *in vitro* environmental conditions. An important addition to the understanding of the SE-stress relationship has been done recently. It is possible to hypothesize that the transition from a vegetative to an embryogenic stage requires a change in the genome organization and, therefore, an active role for chromatin modifications. During the microspore SE in *B. napus*, a decrease in DNA methylation occurs while during the embryo differentiation the DNA methylation increases (Solís et al., 2012). Besides DNA methylation, histone modifications play an important role in the microspore embryogenesis of *B. napus*. Rodríguez-Sanz et al. (2014b) suggest that the marks H3K9me2 and HKMT might participate in the embryo cell differentiation and heterochromatinization and the marks H3Ac, H4Ac, and HAT in events that take place during cell reprogramming and embryo development.

The expression pattern of *BnMET1a*-like genes, which codified for DNA methyltransferases, is highly correlated with the variations in DNA methylation. A DNA hypomethylation in *Quercus suber* has been also documented during the SE from microspores and immature zygotic embryos (Rodríguez-Sanz et al., 2014a). A similar decrease in DNA methylation has been found during the *H. vulgare* microspore SE induction (El-Tantawy et al., 2014). It is worth noting that the SE process in *H. vulgare* is induced with a cold stress instead of a heat stress as happens in *B. napus*, although the response is the same. The transcriptome of *Picea abies* under two epitype-inducing temperatures (18 vs. 30 °C) has also revealed that 35 expressed transcripts, orthologous to epigenetic-related genes, are involved in epigenetic regulation (Yakovlev et al., 2014). These data suggest that temperature-dependent gene expression during the induction of SE could originate from modifications in the chromatin structure.

On the other hand, it was found that explants under embryogenic conditions release organic molecules that inhibit the embryogenic response of somatic cells and also affect DNA methylation levels. There are some reports indicating that phenolic compounds interfere with the SE process (Kouakou et al., 2007; Umehara et al., 2007; Nic-Can et al., 2015) and also inhibit the activity of DNA methyltransferases (Causevic et al., 2005). Recently, it has been shown that *C. arabica* leaves’ explants release several phenolic compounds into the media, which seem to be directly related to the plant’s poor response to direct SE (Nic-Can et al., 2015). Among these compounds, caffeine and chlorogenic acid, which represent 98% of the phenolic compounds, accumulate in the conditioned medium of *C. arabica*. Moreover, the addition of the phenolic compounds, either as conditioned medium or in a pure form, drastically interfers with the SE process in two highly embryogenic species (*C. canephora* and *D. carota*). Global DNA methylation analysis showed that conditioned medium of *C. arabica* stimulates the loss of DNA methylation even more than azaC does. Therefore, the instability of DNA methylation levels because of the accumulation of phenolic compounds could be one of the major causes of the disturbance of cellular metabolism needed to create embryonic complex structures from somatic cells.

## Epigenetic Changes During the Development of Somatic Embryos

In general, variation in DNA methylation is related to developmental changes in response to growth regulator treatments. This methylation is essential during the early development of somatic embryos of *C. pepo* (Leljak-Levanic et al., 2004). The DNA methylation level in *Larix x eurolepis* differs at each step of the development of somatic embryos; it goes from 45.8% in the original embryogenic line to 61.5% after 1 week of maturation. DNA methylation decreases to 53.4% after 8 weeks of maturation (Teyssier et al., 2014). In *C. canephora*, it was also observed that DNA methylation levels increase as the embryo develops; for instance, when the somatic cells of the leaves’ explants begin the cellular dedifferentiation, the content of methylated cytosines is about 23.7%, whereas during the later developmental embryo stages high levels of DNA methylation are established (Nic-Can et al., 2013). On the other hand, the modification of the DNA methylation pattern with azaC or hydroxyurea (hyper-methylating agent) significantly reduced both the relative growth rate and the embryogenic potential (Teyssier et al., 2014).

The treatment of embryogenic lines with a variety of auxin/cytokinin ratios before placement onto a maturation medium containing 40 μM ABA changes the methylation of DNA in the original embryogenic line. The decrease of 2,4-D concentration or its exclusion causes a reduction in the methylation and improves the maturation of somatic embryos in the presence of ABA (Levanic et al., 2009).

Since, in many cases, the elimination of the auxin from the culture medium is necessary for SE to occur, it is possible that the changes in the epigenetic marks form the initial step in the development of somatic embryos. In general the lack of quantitative differences in global cytosine methylation does not necessarily mean that a locus-specific methylation has an important effect on SE induction or development. Thus, more detailed studies are necessary for a deep comprehension of the role of DNA methylation during the induction of SE and the development and transition among the different stages of development of the somatic embryos. One further step in the study of the changes in the epigenetic mechanism is the study of histone marks during the induction of SE.

Recently, a few groups have shown the dynamic activity in the modification of histones that leads to the modulation of the expression of genes that previously have been proposed to be involved in the SE process. For instance, in *C. canephora*, it was found that during the early events of SE the levels of the histone repressive marks H3K9me2 and H3K27me3 decrease, and these events were correlated with the beginning of the expression of *LEC1*, *BBM1* and *WOX4* (Nic-Can et al., 2013). Using chromatin immunoprecipitation (ChIP) assays, it has been found that during the cellular dedifferentiation, the H3K27me3 is removed from *LEC1* loci, allowing the expression of this transcription factor, whereas the expression of *BBM1* was related to the increase of both histone marks H3K4me3 and H3K36me2. In the case of *WOX4*, it was found that its transcriptional repression, especially during the maturation phase of somatic embryos, was correlated with the increase of H3K9me2. This indicates that the chromatin is dynamically regulated to change the transcriptional program of the somatic cells before and during the development of somatic embryos (Nic-Can et al., 2013).

The H3K9me2 mark has also been involved in embryo cell differentiation and heterochromatization events during the microspore embryogenesis in *B. napus* (Rodríguez-Sanz et al., 2014b). Using immunolocalization, it was observed that the levels of H3K9me2 were low in microspores before the induction of SE; however, an increase of more than two times occurs during the late stages of embryogenesis, particularly in the differentiated peripheral cells, indicating a high chromatin condensation. In contrast to H3K9me2, it was observed that the levels of acetylation of H3 and H4 (H3Ac and H4Ac) marks, which are related to transcriptional activity, were more abundant in the microspores before SE induction, especially the H4Ac, suggesting that these modifications might be related to the totipotency acquisition, cellular reprogramming and embryo development. In addition, these patterns were related to changes in the expression profiles of the histone methyltransferase and histone acetyltransferase genes as well as embryogenic development (Rodríguez-Sanz et al., 2014b).

The expression pattern of several genes related to chromatin modification and remodeling [two histone deacetylases (HDACs), *HDA6* and *HDA19*, two histone monoubiquitinases (*HUB1* and *HUB2*), a histone H3 kinase (*AUR3*), *PICKLE* and *VP1*/*ABSCISIC ACID INSNSITIVE 3-LIKE 1* (*VAL1*)], have been studied during the SE process of *Q. suber* (Pérez et al., 2015a). It was found that *QsHDA19* decreases its expression as soon as the callus begins its differentiation, followed by a steady increase from immature cotyledonary embryo to an embryo with the cotyledons fully differentiated. On the other hand, a transient decrease in *QsHDA6*, *QsPICKLE*, and *QsVAL1* gene expression was observed in the transition from callus to the end of the mature embryo. *QsHUB1* and *QsHUB2* showed a transient increase expression from white callogenic structures and globular embryos to immature cotyledonary embryos. The highest expression was observed in white opaque cotyledonary embryos, while *QsAUR3* was preferentially expressed in immature cotyledonary embryos. According to previous reports, histone deacetylases are related to transcriptional repression and chromatin condensation, whereas the monoubiquitination has been associated with transcriptional activation, and the histone kinase is an important mitosis regulator (Turner, 1991; Houben et al., 2005; Cao et al., 2008). In addition, VAL1 and PICKLE are suggested to regulate the repression of the seed transcriptional program (Zhang and Ogas, 2009). All of these results suggest that these epigenetic components play a key role during the development and maturation of *Q. suber* somatic embryos.

Recently, the high resolution of the transcriptome sequencing in *Arabidopsis* has showed that there are important changes in the expression of chromatin-associated genes, which could help to understand the molecular mechanisms that lead to the acquisition of cellular totipotency (Wickramasuriya and Dunwell, 2015). For instance, Chupeau et al. (2013) reported that during the early events of differentiation from protoplast to plantlets a transient up-regulation of histone H3.3 variant occurs due to the incorporation of distinct sets of histone variants in the nucleosomes, particularly because of the enrichment of transcriptional active regions. Moreover, different gene-encoded proteins involved in histone modifications, DNA methylation and demethylation, as well as chromatin remodeling, are also up-regulated, indicating that they play an important role in the overall reprograming of plant cells.

Lately, it has been found that *HDACs* are expressed throughout the SE, whereas histone acetyltransferases accumulate more in somatic embryos than in leaf tissues (Wickramasuriya and Dunwell, 2015). The authors suggest that some members of HDAC family are important for SE in *Arabidopsis*, probably through the regulation of the histone modifications in order to maintain a high methylation status during SE.

Together all of these results suggest that plant chromatin is dynamically regulated during SE, but how the somatic cells break the epigenetic barrier to reach the totipotent status is still a matter of study. It would be interesting to explore how the key genes of the induction of SE are switched on throughout chromatin remodeling in different species or induction conditions. Since stress, like heat in *B. napus* and cold in *H. vulgare*, is one of the inducers of SE, it would be very important to determine whether/how the same epigenetic marks are responsible for both cases.

## Epigenetic Changes in Regenerated Plants

Since the rise of commercial micropropagation, somaclonal variation (SV) has been present as a serious problem, producing many variants among the regenerate plants and, on the other hand, creating a source of variation to achieve new agronomically important cultivars. When the plants come from somatic embryos, the variation can be high. It has been suggested that this variation is due, at least in part, to changes in the DNA methylation pattern. These changes in the DNA methylation of regenerate plants from somatic embryos have been documented in several species. For instance, in maize, a high frequency of DNA methylation variation among regenerates was found (Kaeppler and Phillips, 1993) and, in *Elaeis guineensis*, DNA methylation could be involved in the occurrence of 5% of SV. The DNA methylation present in the leaves of somaclonal regenerates is lower than in the non-variant plants (Jaligot et al., 2000, 2002). Also, the “mantled” SV in somatic embryo-derived oil palm plants (development of abnormal flowers) is associated with a decrease in global DNA methylation (Matthes et al., 2001; Jaligot et al., 2004).This mantle abnormality can be heritable and with time can show reversion to the normal phenotype. The uses of the restriction enzyme Hpall suggest that the loss of methylation occurs most frequently at the internal C (5′-CCGG-3′; Matthes et al., 2001). Regenerated plants from embryogenic callus of *Hordeum brevisubulatum* present a variation frequency of 9.3%. The degree of variation varies among the plants and the variation is present in both protein coding genes. Transposon/retrotransposons were found to underlie the genetic and epigenetic variations (Li et al., 2007).

Furthermore, some factors, such as cryopreservation of the embryogenic tissues, can modify the level of methylation of DNA. It has been determined that plant recovery from cryopreserved somatic embryo clusters of peach palm (*Bactris gasipaes*) showed an increased DNA methylation level when compared with non-cryopreserved somatic embryo clusters. However, 24 weeks after regrowth, the global methylation profile decreases to the initial level (Heringer et al., 2013).

## Conclusion

DNA methylation is an important epigenetic mechanism that has been studied with different approaches. Some of the most used techniques to study DNA methylation have been qualitative and/or quantitative, and the information from each has contributed to the understanding of many important biological questions, such as the mechanisms of SE induction and embryo development. However, it is necessary to explore the role of the different methyltransferases during the SE process, because so far it is poorly understood which of/how these enzymes participate in SE. It is clear that pharmacological assays with azaC have provided some of the answers about whether and how DNA methylation is involved during SE and how it can affect gene expression. The importance of revealing how epigenetics function in SE could help to increase plant productivity and improve agronomical breeding practices.

## Conflict of Interest Statement

The authors declare that the research was conducted in the absence of any commercial or financial relationships that could be construed as a potential conflict of interest.

## References

[B1] Abid G. Muhoviski Y. Jacquemin J. M. Mingeot D. Sassi K. Toussaint A. (2011). Changes in DNA-methylation during zygotic embryogenesis in interspecific hybrids of beans (Phaseolus ssp.). *Plant Cell Tiss. Org. Cult.* 105 383–393. 10.1007/s11240-010-9878-2

[B2] Albert M. Peters A. H. (2009). Genetic and epigenetic control of early mouse development. *Curr. Opin. Genet. Dev.* 19 113–121. 10.1016/j.gde.2009.03.004 19359161

[B3] Amasino R. (2010). Seasonal and developmental timing of flowering. *Plant J.* 61 1001–1013. 10.1111/j.1365-313X.2010.04148.x 20409274

[B4] Baurens F. C. Causse S. Legavre T. (2008). Methylation-sensitive amplification polymorphism (MSAP) protocol to assess CpG and CpNpG methylation in the banana genome. *Fruits* 63 117–123. 10.1051/fruits:2007054

[B5] Belanger F. Hepburn A. (1990). The evolution of CpNpG methylation in plants. *J. Mol. Evol.* 30 26–35. 10.1007/BF02102450

[B6] Bobadilla Landey R. Cenci A. Georget F. Bertrand B. Camayo G. Dechamp E. (2013). High genetic and epigenetic stability in Coffea arabica plants derived from embryogenic suspensions and secondary embryogenesis as revealed by AFLP, MSAP and the phenotypic variation rate. *PLoS ONE* 8:e56372 10.1371/journal.pone.0056372 PMC3572038 23418563

[B7] Bouchard J. Momparler R. L. (1983). Incorporation of 5-Aza-2’-deoxycytidine-5’-triphosphate into DNA. Interactions with mammalian DNA polymerase alpha and DNA methylase. *Mol. Pharmacol.* 24 109–114. 6191192

[B8] Bruce T. Mazur E. Napier J. Pickett J. (2007). Stressful “memories” of plants: evidence and possible mechanisms. *Plant Sci.* 173 603–608. 10.1016/j.plantsci.2007.09.002

[B9] Cabrera-Ponce J. L. López L. León-Ramírez C. G. Jofre-Garfias A. E. Vargas A. (2015). Stress induced acquisition of somatic embryogenesis in common bean *Phaseolus vulgaris* L. *Protoplasma* 252 559–570. 10.1007/s00709-014-0702-4 25252886

[B10] Cao X. Jacobsen S. E. (2002). Locus-specific control of asymmetric and CpNpG methylation by the DRM and CMT3 methyltransferase genes. *Proc. Natl. Acad. Sci. U.S.A.* 99 16491–16498. 10.1073/pnas.162371599 12151602 PMC139913

[B11] Cao Y. Dai Y. Cui S. Ma L. (2008). Histone H2B monoubiquitination in the chromatin of FLOWERING LOCUS C regulates flowering time in *Arabidopsis*. *Plant Cell* 20 2586–2602. 10.1105/tpc.108.062760 18849490 PMC2590739

[B12] Causevic A. Delaunay A. Ounnar S. Righezza M. Delmotte F. Brignolas F. (2005). DNA methylating and demethylating treatments modify phenotype and cell wall differentiation state in sugarbeet cell lines. *Plant Physiol. Biochem.* 43 681–691. 10.1016/j.plaphy.2005.05.011 16046142

[B13] Chakrabarty D. Yu K. W. Paek K. Y. (2003). Detection of DNA methylation changes during somatic embryogenesis of Siberian ginseng (*Eleuterococcus senticosus*). *Plant Sci.* 165 61–68. 10.1016/S0168-9452(03)00127-4

[B14] Chupeau M. C. Granier F. Pichon O. Renou J. P. Gaudin V. Chupeau Y. (2013). Characterization of the early events leading to totipotency in an *Arabidopsis* protoplast liquid culture by temporal transcript profiling. *Plant Cell* 25 2444–2463. 10.1105/tpc.113.109538 23903317 PMC3753376

[B15] Cokus S. J. Feng S. Zhang X. Chen Z. Merriman B. Haudenschild C. D. (2008). Shotgun bisulphite sequencing of the *Arabidopsis* genome reveals DNA methylation patterning. *Nature* 452 215–219. 10.1038/nature06745 18278030 PMC2377394

[B16] Cox R. Irving C. C. (1977). Inhibition of DNA methylation by S-adenosylethionine with the production of methyl-deficient DNA in regenerating rat liver. *Cancer Res.* 37 222–225. 830409

[B17] Cristofolini C. do Nascimento Vieira L. de Freitas Fraga H. da Costa I. Guerra M. Pescador R. (2014). DNA methylation patterns and karyotype analysis of off-type and normal phenotype somatic embryos of feijoa. *Theor. Exp. Plant Physiol.* 26 217–224. 10.1007/s40626-014-0020-4

[B18] Decout E. Dubois T. Guedira M. Dubois J. Audran J. C. Vasseur J. (1994). Role of temperature as a triggering signal for organogenesis or somatic embryogenesis in wounded leaves of chicory cultured in vitro. *J. Exp. Bot.* 45 1859–1865. 10.1093/jxb/45.12.1859

[B19] De-la-Peña C. Nic-Can G. Ojeda G. Herrera-Herrera J. Lopez-Torres A. Wrobel K. (2012). KNOX1 is expressed and epigenetically regulated during in vitro conditions in *Agave* spp. *BMC Plant Biol.* 12:203 10.1186/1471-2229-12-203 PMC3541254 23126409

[B20] D’Incalci M. Covey J. M. Zaharko D. S. Kohn K. W. (1985). DNA alkali-labile sites induced by incorporation of 5-aza-2’-deoxycytidine into DNA of mouse leukemia L1210 cells. *Cancer Res.* 45 3197–3202. 2408746

[B21] Du J. Zhong X. Bernatavichute Y. V. Stroud H. Feng S. Caro E. (2012). Dual binding of chromomethylase domains to H3K9me2-containing nucleosomes directs DNA methylation in plants. *Cell* 151 167–180. 10.1016/j.cell.2012.07.034 23021223 PMC3471781

[B22] El-Tantawy A. A. Solís M. T. Risueño M. C. Testillano P. S. (2014). Changes in DNA methylation levels and nuclear distribution patterns after microspore reprogramming to embryogenesis in barley. *Cytogenet. Genome Res.* 143 200–208. 10.1159/000365232 25074410

[B23] Fehér A. (2015). Somatic embryogenesis - Stress-induced remodeling of plant cell fate. *BBA Gene Regul. Mech.* 1849 385–402. 10.1016/j.bbagrm.2014.07.005 25038583

[B24] Feng S. Cokus S. J. Zhang X. Chen P.-Y. Bostick M. Goll M. G. (2010). Conservation and divergence of methylation patterning in plants and animals. *Proc. Natl. Acad. Sci. U.S.A.* 107 8689–8694. 10.1073/pnas.1002720107 20395551 PMC2889301

[B25] Feng S. Jacobsen S. E. (2011). Epigenetic modifications in plants: an evolutionary perspective. *Curr. Opin. Plant Biol.* 14 179–186. 10.1016/j.pbi.2010.12.002 21233005 PMC3097131

[B26] Finnegan E. J. Genger R. K. Peacock W. J. Dennis E. S. (1998). DNA methylation in plants. *Annu. Rev. Plant Physiol. Plant Mol. Biol.* 49 223–247. 10.1146/annurev.arplant.49.1.223 15012234

[B27] Finnegan E. J. Kovac K. A. (2000). Plant DNA methyltransferases. *Plant Mol. Biol.* 43 189–201. 10.1023/A:1006427226972 10999404

[B28] Fraga H. P. F. Vieira L. N. Caprestano C. A. Steinmacher D. A. Micke G. A. Spudeit D. A. (2012). 5-Azacytidine combined with 2,4-D improves somatic embryogenesis of *Acca sellowiana* (O. Berg) Burret by means of changes in global DNA methylation levels. *Plant Cell Rep.* 31 2165–2176. 10.1007/s00299-012-1327-8 22865112

[B29] Fraga M. F. Rodríguez R. Cañal M. J. (2000). Rapid quantification of DNA methylation by high performance capillary electrophoresis. *Electrophoresis* 21 2990–2994. 10.1002/1522-2683(20000801)21:14<2990::AID-ELPS2990>3.3.CO;2-9 11001314

[B30] Frommer M. McDonald L. E. Millar D. S. Collis C. M. Watt F. Grigg G. W. (1992). A genomic sequencing protocol that yields a positive display of 5-methylcytosine residues in individual DNA strands. *Proc. Natl. Acad. Sci. U.S.A.* 89 1827–1831. 10.1073/pnas.89.5.1827 1542678 PMC48546

[B31] Fuentes-Cerda C. F. J. Monforte-González M. Méndez-Zeel M. Rojas-Herrera R. Loyola-Vargas V. M. (2001). Modification of the embryogenic response of *Coffea arabica* by nitrogen source. *Biotechnol. Lett.* 23 1341–1343. 10.1023/A:1010545818671

[B32] Goffin J. Eisenhauer E. (2002). DNA methyltransferase inhibitors - state of the art. *Ann. Oncol.* 13 1699–1716. 10.1093/annonc/mdf314 12419742

[B33] Gonzalo S. (2010). Epigenetic alterations in aging. *J. Appl. Physiol.* 109 586–597. 10.1152/japplphysiol.00238.2010 20448029 PMC2928596

[B34] Grace Goll M. Bestor T. H. (2005). Eukaryotic cytosine methyltransferases. *Annu. Rev. Biochem.* 74 481–514. 10.1146/annurev.biochem.74.010904.153721 15952895

[B35] Grigg G. W. (1996). Sequencing 5-methylcytosine residues by the bisulphite method. *DNA Seq.* 6 189–198. 10.3109/10425179609008443 8912921

[B36] Gruenbaum Y. Naveh-Many T. Cedar H. Razin A. (1981). Sequence specificity of methylation in higher plant DNA. *Nature* 292 860–862. 10.1038/292860a0 6267477

[B37] Hajkova P. El-Maarri O. Engemann S. Oswald J. Olek A. Walter J. (2002). “DNA-methylation analysis by the bisulfite-assisted genomic sequencing method”, in *DNA Methylation Protocols*, eds Mills K. Ramsahoye B. (New York: Springer), 143–154. 10.1385/1-59259-182-5:143 11951649

[B38] Henikoff S. Comai L. (1998). A DNA methyltransferase homolog with a chromodomain exists in multiple polymorphic forms in *Arabidopsis. Genetics* 149 307–318. 9584105 10.1093/genetics/149.1.307 PMC1460135

[B39] Heringer A. S. Steinmacher D. A. Fraga H. P. Vieira L. N. Ree J. F. Guerra M. P. (2013). Global DNA methylation profiles of somatic embryos of peach palm (*Bactris gasipaes Kunth*) are influenced by cryoprotectants and droplet-vitrification cryopreservation. *Plant Cell Tiss. Org. Cult.* 114 365–372. 10.1007/s11240-013-0331-1

[B40] Hirochika H. Okamoto H. Kakutani T. (2000). Silencing of retrotransposons in *Arabidopsis* and reactivation by the ddm1 mutation. *Plant Cell* 12 357–368. 10.1105/tpc.12.3.357 10715322 PMC139836

[B41] Houben A. Demidov D. Rutten T. Scheidtmann K. H. (2005). Novel phosphorylation of histone H3 at threonine 11 that temporally correlates with condensation of mitotic and meiotic chromosomes in plant cells. *Cytogenet. Genome Res.* 109 148–155. 10.1159/000082394 15753571

[B42] Ikeda-Iwai M. Umehara M. Satoh S. Kamada H. (2003). Stress-induced somatic embryogenesis in vegetative tissues of *Arabidopsis thaliana*. *Plant J.* 34 107–114. 10.1046/j.1365-313X.2003.01702.x 12662313

[B43] Jaligot E. Beulé T. Baurens F. C. Billotte N. Rival A. (2004). Search for methylation-sensitive amplification polymorphisms associated with the “mantled” variant phenotype in oil palm (*Elaeis guineensis Jacq*.). *Genome* 47 224–228. 10.1139/g03-085 15060619

[B44] Jaligot E. Beulé T. Rival A. (2002). Methylation-sensitive RFLPs: characterisation of two oil palm markers showing somaclonal variation-associated polymorphism. *Theor. Appl. Genet.* 104 1263–1269. 10.1007/s00122-002-0906-4 12582579

[B45] Jaligot E. Rival A. Beulé T. Dussert S. Verdeil J. L. (2000). Somaclonal variation in oil palm (*Elaeis guineensis Jacq*.): the DNA methylation hypothesis. *Plant Cell Rep.* 19 684–690. 10.1007/s002999900177 30754806

[B46] Jean Finnegan E. Dennis E. S. (1993). Isolation and identification by sequence homology of a putative cytosine methyltransferase from *Arabidopsis thaliana*. *Nucleic Acids Res.* 21 2383–2388. 10.1093/nar/21.10.2383 8389441 PMC309536

[B47] Jin F. Hu L. Yuan D. Xu J. Gao W. He L. (2014). Comparative transcriptome analysis between somatic embryos (SEs) and zygotic embryos in cotton: evidence for stress response functions in SE development. *Plant Biotechnol. J.* 12 161–173. 10.1111/pbi.12123 24112122

[B48] Jones P. A. Taylor S. M. (1980). Cellular differentiation, cytidine analogs and DNA methylation. *Cell* 20 85–93. 10.1016/0092-8674(80)90237-8 6156004

[B49] Jüttermann R. Li E. Jaenisch R. (1994). Toxicity of 5-aza-2’-deoxycytidine to mammalian cells is mediated primarily by covalent trapping of DNA methyltransferase rather than DNA demethylation. *Proc. Natl. Acad. Sci. U.S.A.* 91 11797–11801. 10.1073/pnas.91.25.11797 7527544 PMC45322

[B50] Kaeppler S. M. Phillips R. L. (1993). Tissue culture-induced DNA methylation variation in maize. *Proc. Natl. Acad. Sci. U.S.A.* 90 8773–8776. 10.1073/pnas.90.19.8773 8415605 PMC47442

[B51] Kamada H. Ishikawa K. Saga H. Harada H. (1993). Induction of somatic embryogenesis in carrot by osmotic stress. *Plant Tiss. Cult. Lett.* 10 38–44. 10.5511/plantbiotechnology1984.10.38

[B52] Kamada H. Tachikawa Y. Saitou T. Harada H. (1994). Heat stress induction of carrot somatic embryogenesis. *Plant Tiss. Cult. Lett.* 11 229–232. 10.5511/plantbiotechnology1984.11.229

[B53] Kathiria P. Kovalchuk I. (2010). “In situ analysis of DNA methylation in plants,” in *Plant Epigenetics*, eds Kovalchuk I. Zemp F. J. (London: Humana Press), 41–48. 10.1007/978-1-60761-646-7_5

[B54] Kato M. Miura A. Bender J. Jacobsen S. E. Kakutani T. (2003). Role of CG and non-CG methylation in immobilization of transposons in *Arabidopsis*. *Curr. Biol* 13 421–426. 10.1016/S0960-9822(03)00106-4 12620192

[B55] Klimasauskas S. Kumar S. Roberts R. J. Cheng X. (1994). Hhal methyltransferase flips its target base out of the DNA helix. *Cell* 76 357–369. 10.1016/0092-8674(94)90342-5 8293469

[B56] Klimaszewska K. Noceda C. Pelletier G. Label P. Rodriguez R. Lelu-Walter M. A. (2009). Biological characterization of young and aged embryogenic cultures of *Pinus pinaster* (Ait.). *In Vitro Cell. Dev. Biol. Plant* 45 20–33. 10.1007/s11627-008-9158-6

[B57] Köhler C. Wolff P. Spillane C. (2012). Epigenetic mechanisms underlying genomic imprinting in plants. *Annu. Rev. Plant Biol.* 63 331–352. 10.1146/annurev-arplant-042811-105514 22404470

[B58] Kouakou T. H. Waffo-Téguo P. Kouadio Y. J. VAlls J. Richard T. Decendit A. (2007). Phenolic compounds and somatic embryogenesis in cotton (*Gossypium hirsutum* L.). *Plant Cell Tiss. Org. Cult.* 90 25–29. 10.1007/s11240-007-9243-2

[B59] Kovalchuk I. Zemp F. J. (2010). *Plant Epigenetics. Methods and Protocols.* London: Springer.

[B60] Law J. A. Jacobsen S. E. (2010). Establishing, maintaining and modifying DNA methylation patterns in plants and animals. *Nat. Rev. Genet.* 11 204–220. 10.1038/nrg2719 20142834 PMC3034103

[B61] Leljak-Levanic D. Bauer N. Mihaljevic S. Jelaska S. (2004). Changes in DNA methylation during somatic embryogenesis in *Cucurbita pepo* L. *Plant Cell Rep.* 23 120–127. 10.1007/s00299-004-0819-6 15221278

[B62] Levanic D. L. Mihaljevic S. Jelaska S. (2009). Variations in DNA methylation in Picea Omorika (Panc) Purk. embryogenic tissue and the ability for embryo maturation. *Prop. Orn. Plants* 9 3–9.

[B63] Li X. Yu X. Wang N. Feng Q. Dong Z. Liu L. (2007). Genetic and epigenetic instabilities induced by tissue culture in wild barley (*Hordeum brevisubulatum* (Trin.) Link). *Plant Cell Tiss. Org. Cult.* 90 153–168. 10.1007/s11240-007-9224-5

[B64] Li Y. Shan X. Liu X. Hu L. Guo W. Liu B. (2008). Utility of the methylation-sensitive amplified polymorphism (MSAP) marker for detection of DNA methylation polymorphism and epigenetic population structure in a wild barley species (*Hordeum brevisubulatum*). *Ecol. Res.* 23 927–930. 10.1007/s11284-007-0459-8

[B65] Lin K. T. Momparlerm R. L. Rivard G. E. (1981). High-performance liquid chromatographic analysis of chemical stability of 5-aza-2’-deoxycytidine. *J. Pharmaceut. Sci.* 70 1228–1232. 10.1002/jps.2600701112 6170748

[B66] Lindroth A. M. Cao X. Jackson J. P. Zilberman D. McCallum C. M. Henikoff S. (2001). Requirement of CHROMOMETHYLASE3 for maintenance of CpXpG methylation. *Science* 292 2077–2080. 10.1126/science.1059745 11349138

[B67] Lister R. O’Malley R. C. Tonti-Filippini J. Gregory B. D. Berry C. C. Millar A. H. (2008). Highly integrated single-base resolution maps of the epigenome in *Arabidopsis*. *Cell* 133 523–536. 10.1016/j.cell.2008.03.029 18423832 PMC2723732

[B68] Liutkeviciute Z. Kriukiene E. Grigaityte I. Masevicius V. Klimasauskas S. (2011). Methyltransferase-directed derivatization of 5-hydroxymethylcytosine in DNA. *Angew. Chem.* 123 2138–2141. 10.1002/ange.201007169 PMC3137911 21344558

[B69] Lopez Torres A. Yanez Barrientos E. Wrobel K. Wrobel K. (2011). Selective derivatization of cytosine- and methylcytosine moieties with 2-bromoacetonephenone for sub-microgram DNA methylation analysis by reversed phase HPLC with spectrofluorimetric detection. *Anal. Chem.* 83 7999–8005. 10.1021/ac2020799 21905673

[B70] LoSchiavo F. Pitto L. Giuliano G. Torti G. Nuti-Ronchi V. Marazziti D. (1989). DNA methylation of embryogenic carrot cell cultures and its variations as caused by mutation, differentiation, hormones and hypomethylating drugs. *Theor. Appl. Genet.* 77 325–331. 10.1007/BF00305823 24232608

[B71] Loyola-Vargas V. M. De-la-Peña C. Galaz-Avalos R. M. Quiroz-Figueroa F. R. (2008). “Plant tissue culture. An intemporal set of tools,” in *Protein and Cell Biomethods Handbook*, eds Walker J. M. Rapley R. (Totowa: Humana Press), 875–904. 10.1007/978-1-60327-375-6_50

[B72] Lübbert M. (2000). DNA methylation inhibitors in the treatment of leukemias, myelodysplastic syndromes and hemoglobinopathies: clinical results and possible mechanisms of action. *Curr. Top. Microbiol. Immunol.* 249 135–164. 10802943 10.1007/978-3-642-59696-4_9

[B73] Magaña Alcázar A. Wrobel K. Alvarado Caudillo Y. Zaina S. Lund G. Wrobel K. (2008). High-performance liquid chromatography determination of 5-methyl-2’-deoxycytidine, 2’-deoxycytidine, and other deoxynucleosides and nucleosides in DNA digests. *Anal. Biochem.* 374 378–385. 10.1016/j.ab.2007.11.026 18157934

[B74] Martienssen R. A. Colot V. (2001). DNA methylation and epigenetic inheritance in plants and filamentous fungi. *Science* 293 1070–1074. 10.1126/science.293.5532.1070 11498574

[B75] Matthes M. Singh R. Cheah S. C. Karp A. (2001). Variation in oil palm (*Elaeis guineensis* Jacq.) tissue culture-derived regenerants revealed by AFLPs with methylation-sensitive enzymes. *Theor. Appl. Genet.* 102 971–979. 10.1007/s001220000491

[B76] Meijón M. Valledor L. Santamaría E. Testillano P. S. Risueño M. C. Rodríguez R. (2009). Epigenetic characterization of the vegetative and floral stages of azalea buds: dynamics of DNA methylation and histone H4 acetylation. *J. Plant Physiol.* 166 1624–1636. 10.1016/j.jplph.2009.04.014 19523713

[B77] Meneses A. Flores D. Muñoz M. Arrieta G. Espinoza A. M. (2010). Effect of 2,4-D, hydric stress and light on indica rice (*Oryza sativa*) somatic embryogenesis. *Rev. Biol. Trop.* 53 361–368. 10.15517/rbt.v53i3-4.14598 17354447

[B78] Miguel C. Marum L. (2011). An epigenetic view of plant cells cultured in vitro: somaclonal variation and beyond. *J. Exp. Bot.* 62 3713–3725. 10.1093/jxb/err155 21617249

[B79] Mihaljevic S. Radic S. Bauer N. Garic R. Mihaljevic B. Horvat G. (2011). Ammonium-related metabolic changes affect somatic embryogenesis in pumpkin (*Cucurbita pepo* L.). *J. Plant Physiol.* 168 1943–1951. 10.1016/j.jplph.2011.05.025 21807439

[B80] Monk M. Boubelik M. Lehnert S. (1987). Temporal and regional changes in DNA methylation in the embryonic, extraembryonic and germ cell lineages during mouse embryo development. *Development* 99 371–382. 3653008 10.1242/dev.99.3.371

[B81] Neelakandan A. Wang K. (2012). Recent progress in the understanding of tissue culture-induced genome level changes in plants and potential applications. *Plant Cell Rep.* 31 597–620. 10.1007/s00299-011-1202-z 22179259

[B82] Nic-Can G. I. De-la-Peña C. (2014). “Epigenetic advances on somatic embryogenesis of agronomical and important crops,” in *Epigenetics in Plants of Agronomic Importance: Fundamentals and Applications*, eds Álvarez-Venegas R. De-la-Peña C. Casas-Mollano J. A. (London: Springer), 91–109. 10.1007/978-3-319-07971-4_6

[B83] Nic-Can G. I. Galaz-Ávalos R. M. De-la-Peña C. Alcazar-Magaña A. Wrobel K. Loyola-Vargas V. M. (2015). Somatic embryogenesis: identified factors that lead to embryogenic repression. A case of species of the same genus. *PLoS ONE* 10:e0126414 10.1371/journal.pone.0126414 PMC4454440 26038822

[B84] Nic-Can G. I. López-Torres A. Barredo-Pool F. A. Wrobel K. Loyola-Vargas V. M. Rojas-Herrera R. (2013). New insights into somatic embryogenesis: LEAFY COTYLEDON1, BABY BOOM1 and WUSCHEL-RELATED HOMEOBOX4 are epigenetically regulated in Coffea canephora. *PLoS ONE* 8:e72160 10.1371/journal.pone.0072160 PMC3748027 23977240

[B85] Noceda C. Salaj T. Pérez M. Viejo M. Cañal J. Salaj J. (2009). DNA methylation and decrease on free polyamines is associated with the embryogenic capacity of *Pinus nigra* Arn. *Cell Cult. Trees* 23 1285–1293. 10.1007/s00468-009-0370-8

[B86] Nodine M. D. Bartel D. P. (2010). MicroRNAs prevent precocious gene expression and enable pattern formation during plant embryogenesis. *Gene. Dev.* 24 2678–2692. 10.1101/gad.1986710 21123653 PMC2994041

[B87] Nolan K. E. Saeed N. A. Rose R. J. (2006). The stress kinase gene MtSK1 in *Medicago truncatula* with particular reference to somatic embryogenesis. *Plant Cell Rep.* 25 711–722. 10.1007/s00299-006-0135-4 16518633

[B88] Okano M. Bell D. W. Haber D. A. Li E. (1999). DNA methyltransferases Dnmt3a and Dnmt3b are essential for de novo methylation and mammalian development. *Cell* 99 247–257. 10.1016/S0092-8674(00)81656 10555141

[B89] Palmgren G. Mattsson O. Okkels F. T. (1991). Specific levels of DNA methylation in various tissues, cell lines, and cell types of *Daucus carota*. *Plant Physiol.* 95 174–178. 10.1104/pp.95.1.174 16667947 PMC1077502

[B90] Pasternak T. P. Prinsen E. Ayaydin F. Miskolczi P. Potters G. Asard H. (2002). The role of auxin, pH, and stress in the activation of embryogenic cell division in leaf protoplast-derived cells of alfalfa. *Plant Physiol.* 129 1807–1819. 10.1104/pp.000810 12177494 PMC166769

[B91] Patnaik D. Mahalakshmi A. Khurana P. (2005). Effect of water stress and heavy metals on induction of somatic embryogenesis in wheat leaf base cultures. *Indian J. Exp. Biol.* 43 740–755. 16121718

[B92] Peräkylä M. (1998). A model study of the enzyme-catalyzed cytosine methylation using ab initio quantum mechanical and density functional theory calculations: pKa of the cytosine N3 in the intermediates and transition states of the reaction. *J. Am. Chem. Soc.* 120 12895–12902. 10.1021/ja981405a

[B93] Pérez M. Cañal M. J. Toorop P. E. (2015a). Expression analysis of epigenetic and abscisic acid-related genes during maturation of *Quercus suber* somatic embryos. *Plant Cell Tiss. Org. Cult.* 121 353–366. 10.1007/s11240-014-0706-y

[B94] Pérez M. Viejo M. LaCuesta M. Toorop P. Cañal M. J. (2015b). Epigenetic and hormonal profile during maturation of *Quercus suber* L. *somatic embryos.* *J. Plant Physiol.* 173 51–61. 10.1016/j.jplph.2014.07.028 25462078

[B95] Potters G. Pasternak T. P. Guisez Y. Palme K. J. Jansen M. A. K. (2007). Stress-induced morphogenic responses: growing out of trouble? *Trends Plant Sci.* 12 98–105. 10.1016/j.tplants.2007.01.004 17287141

[B96] Raugei G. Bazzicalupo M. Federici G. Gallori E. Pepino R. Polsinelli M. (1981). Effect of a new pyrimidine analog on *Bacillus subtilis* growth. *J. Bacteriol.* 145 1079–1081. 6780528 10.1128/jb.145.2.1079-1081.1981 PMC217220

[B97] Rein T. DePamphilis M. L. Zorbas H. (1998). Identifying 5-methylcytosine and related modifications in DNA genomes. *Nucleic Acids Res.* 26 2255–2264. 10.1093/nar/26.10.2255 9580672 PMC147551

[B98] Reyna-López G. E. Simpson J. Ruiz-Herrera J. (1997). Differences in DNA methylation patterns are detectable during the dimorphic transition of fungi by amplification of restriction polymorphisms. *Mol. Gen. Genet.* 253 703–710. 10.1007/s004380050374 9079881

[B99] Rodríguez-Sanz H. Manzanera J. A. Solís M. T. Gómez-Garay A. Pintos B. Risueño M. C. (2014a). Early markers are present in both embryogenesis pathways from microspores and immature zygotic embryos in cork oak, *Quercus suber* L. *BMC Plant Biol.* 14:224 10.1186/s12870-014-0224-4 PMC4147960 25162300

[B100] Rodríguez-Sanz H. Moreno-Romero J. Solís M. T. Köhler C. Risueño M. C. Testllano P. S. (2014b). Changes in histone methylation and acetylation during microspore reprogramming to embryogenesis occur concomitantly with BnHKMT and BnHAT expression and are associated to cell totipotency, proliferation and differentiation in *Brassica napus*. *Cytogenet. Genome Res.* 143 209–218. 10.1159/000365261 25060767

[B101] Salvo S. A. G. D. Hirsch C. N. Buell C. R. Kaeppler S. M. Kaeppler H. F. (2014). Whole transcriptome profiling of maize during early somatic embryogenesis reveals altered expression of stress factors and embryogenesis-related genes. *PLoS ONE* 9:e111407 10.1371/journal.pone.0111407 PMC4214754 25356773

[B102] Santi D. V. Garret C. E. Barr P. J. (1983). On the mechanism of inhibition of DNA-cytosine methyltransferases by cytosine analogs. *Cell* 33 9–10. 10.1016/0092-8674(83)90327-6 6205762

[B103] Santi D. V. Norment A. Garrett C. E. (1984). Covalent bond formation between a DNA-cytosine methyltransferase and DNA containing 5-azacytosine. *Proc. Natl. Acad. Sci. U.S.A.* 81 6993–6997. 10.1073/pnas.81.22.6993 6209710 PMC392062

[B104] Santini V. Kantarjian H. Issa J.-P. (2001). Changes in DNA methylation in neoplasia: pathophysiology and therapeutic implications. *Ann. Int. Med.* 134 573–586. 10.7326/0003-4819-134-7-200104030-00011 11281740

[B105] Santos D. Fevereiro P. (2002). Loss of DNA methylation affects somatic embryogenesis in *Medicago truncatula*. *Plant Cell Tiss. Org. Cult.* 70 155–161. 10.1023/A:1016369921067

[B106] Schones D. E. Zhao K. (2008). Genome-wide approaches to studying chromatin modifications. *Nat. Rev. Genet.* 9 179–191. 10.1038/nrg2270 18250624 PMC10882563

[B107] Shibukawa T. Yazawa K. Kikuchi A. Kamada H. (2009). Possible involvement of DNA methylation on expression regulation of carrot LEC1 gene in its 5’-upstream region. *Gene* 437 22–31. 10.1016/j.gene.2009.02.011 19264116

[B108] Singer T. Yordan C. Martienssen R. A. (2001). Robertson’s mutator transposons in A. thaliana are regulated by the chromatin-remodeling gene decrease in DNA Methylation (DDM1). *Gene. Dev.* 15 591–602. 10.1101/gad.193701 11238379 PMC312647

[B109] Smykal P. Valledor L. Rodríguez R. Griga M. (2007). Assessment of genetic and epigenetic stability in long-term in vitro shoot culture of pea (*Pisum sativum* L.). *Plant Cell Rep.* 26 1985–1998. 10.1007/s00299-007-0413-9 17668220

[B110] Solís M. T. El-Tantawy A. A. Cano V. Risueño M. C. Testillano P. S. (2015). 5-azacytidine promotes microspore embryogenesis initiation by decreasing global DNA methylation, but prevents subsequent embryo development in rapeseed and barley. *Front. Plant Sci.* 6:472 10.3389/fpls.2015.00472 PMC4479788 26161085

[B111] Solís M. T. Rodríguez-Serrano M. Meijón M. Cañal M. J. Cifuentes A. Risueño M. C. (2012). DNA methylation dynamics and MET1α-like gene expression changes during stress-induced pollen reprogramming to embryogenesis. *J. Exp. Bot.* 63 6431–6444. 10.1093/jxb/ers298 23175669 PMC3504494

[B112] Spillane C. McKeown P. C. (2014). *Plant Epigenetics and Epigenomics.* London: Springer.

[B113] Tamaru H. (2010). Confining euchromatin/heterochromatin territory: jumonji crosses the line. *Gene. Dev.* 24 1465–1478. 10.1101/gad.1941010 20634313 PMC2904936

[B114] Tamura K. Nei M. (1993). Estimation of the number of nucleotide substitutions in the control region of mitochondrial DNA in humans and chimpanzees. *Mol. Biol. Evol.* 10 512–526. 8336541 10.1093/oxfordjournals.molbev.a040023

[B115] Tamura K. Stecher G. Peterson D. Filipski A. Kumar S. (2013). MEGA6: molecular evolutionary genetics analysis version 6.0. *Mol. Biol. Evol.* 30 2725–2729. 10.1093/molbev/mst197 24132122 PMC3840312

[B116] Testillano P. S. Solís M. T. Risueño M. C. (2013). The 5-methyl-deoxy-cytidine (5mdC) localization to reveal in situ the dynamics of DNA methylation chromatin pattern in a variety of plant organ and tissue cells during development. *Physiol. Plant* 149 104–113. 10.1111/ppl.12015 23193951

[B117] Teyssier C. Maury S. Beaufour M. Grondin C. Delaunay A. Le Metté C. (2014). In search of markers for somatic embryo maturation in hybrid larch (Larix x eurolepis): global DNA methylation and proteomic analyses. *Physiol. Plant* 150 271–291. 10.1111/ppl.12081 23789891

[B118] Tollefsbo T. O. (2011). *Epigenetics Protocols.* London: Springer.

[B119] Tost J. (2009). *DNA Methylation. Methods and Protocols.* New York: Humana Press.

[B120] Touraev A. Ilham A. Vicente O. Heberle-Bors E. (1996). Stress-induced microspore embryogenesis in tobacco: an optimized system for molecular studies. *Plant Cell Rep.* 15 561–565. 10.1007/BF00232453 24178518

[B121] Tsukahara S. Kobayashi A. Kawabe A. Mathieu O. Miura A. Kakutani T. (2009). Bursts of retrotransposition reproduced in *Arabidopsis*. *Nature* 461 423–426. 10.1038/nature08351 19734880

[B122] Turner B. M. (1991). Histone acetylation and control of gene expression. *J. Cell Sci.* 99 13–20. 1757496 10.1242/jcs.99.1.13

[B123] Umehara M. Ikeda M. Kamada H. (2007). Endogenous factors that regulate plant embryogenesis: recent advances. *Jap. J. Plant Sci.* 1 1–6.

[B124] Us-Camas R. Rivera-Solís G. Duarte-Aké F. De-la-Peña C. (2014). In vitro culture: an epigenetic challenge for plants. *Plant Cell Tiss. Org. Cult.* 118 187–201. 10.1007/s11240-014-0482-8

[B125] Valledor L. Hasbún R. Meijón M. Rodríguez J. Santamaría E. Viejo M. (2007). Involvement of DNA methylation in tree development and micropropagation. *Plant Cell Tiss. Org. Cult.* 91 75–86. 10.1007/s11240-007-9262-z

[B126] Vanyushin B. F. (1984). Replicative DNA methylation in animals and higher plants. *Curr. Top. Microbiol. Immunol.* 108 99–114. 6370615 10.1007/978-3-642-69370-0_7

[B127] Viejo M. Rodríguez R. Valledor L. Pérez M. Cañal M. Hasbún R. (2010). DNA methylation during sexual embryogenesis and implications on the induction of somatic embryogenesis in *Castanea sativa* Miller. *Sex. Plant Reprod.* 23 315–323. 10.1007/s00497-010-0145-9 20552230

[B128] Wang R. Y. H. Gehrke C. W. Ehrlich M. (1980). Comparison of bisulfite modification of 5-methyldeoxycytidine and deoxycytidine residues. *Nucleic Acids Res.* 8 4777–4790. 10.1093/nar/8.20.4777 7443525 PMC324387

[B129] Wickramasuriya A. M. Dunwell J. M. (2015). Global scale transcriptome analysis of *Arabidopsis* embryogenesis in vitro. *BMC Genomics* 16:301 10.1186/s12864-015-1504-6 PMC4404573 25887996

[B130] Wollmann H. Berger F. (2012). Epigenetic reprogramming during plant reproduction and seed development. *Curr. Opi. Plant Biol.* 15 63–69. 10.1016/j.pbi.2011.10.001 22035873

[B131] Wolny E. Braszewska-Zalewska A. Hasterok R. (2014). Spatial distribution of epigenetic modifications in *Brachypodium distachyon* embryos during seed maturation and germination. *PLoS ONE* 9:e101246 10.1371/journal.pone.0101246 PMC4090163 25006668

[B132] Wrobel K. Wrobel K. Caruso J. (2009). Epigenetics: an important challenge for ICP-MS in metallomics studies. *Anal. Bioanal. Chem* 393 481–486. 10.1007/s00216-008-2472-3 18979091

[B133] Wu J. C. Santi D. V. (1987). Kinetic and catalytic mechanism of HhaI methyltransferase. *J. Biol. Chem.* 262 4778–4786. 3558369

[B134] Xu M. Li X. Korban S. (2004). DNA-methylation alterations and exchanges during in vitro cellular differentiation in rose (*Rosa hybrida* L.). *Theor. Appl. Genet.* 109 899–910. 10.1007/s00122-004-1717-6 15221146

[B135] Yakovlev I. A. Lee Y. Rotter B. Olsen J. E. Skroppa T. Johnsen O. (2014). Temperature-dependent differential transcriptomes during formation of an epigenetic memory in Norway spruce embryogenesis. *Tree Gen. Genom.* 10 355–366. 10.1007/s11295-013-0691-z

[B136] Yamamoto N. Kobayashi H. Togashi T. Mori Y. Kikuchi K. Kuriyama K. (2005). Formation of embryogenic cell clumps from carrot epidermal cells is suppressed by 5-azacytidine, a DNA methylation inhibitor. *J. Plant Physiol.* 162 47–54. 10.1016/j.jplph.2004.05.013 15700420

[B137] Yang X. Zhang X. (2010). Regulation of somatic embryogenesis in higher plants. *Crit. Rev. Plant Sci.* 29 36–57. 10.1080/07352680903436291

[B138] Zhang H. Ogas J. (2009). An epigenetic perspective on developmental regulation of seed genes. *Mol. Plant* 2 610–627. 10.1093/mp/ssp027 19825643

[B139] Zhao J. P. Simmonds D. H. Newcomb W. (1996). High frequency production of doubled haploid plants of *Brassica napus cv Topas* derived from colchicine-induced microspore embryogenesis without heat shock. *Plant Cell Rep.* 15 668–671. 10.1007/BF00231921 24178607

[B140] Zhong X. Du J. Hale C. J. Gallego-Bartolome J. Feng S. Vashisht A. A. (2014). Molecular mechanism of action of plant DRM de novo DNA methyltransferases. *Cell* 157 1050–1060. 10.1016/j.cell.2014.03.056 24855943 PMC4123750

